# Density-dependence interacts with extrinsic mortality in shaping life histories

**DOI:** 10.1371/journal.pone.0186661

**Published:** 2017-10-19

**Authors:** Maciej Jan Dańko, Oskar Burger, Jan Kozłowski

**Affiliations:** 1 Max Planck Institute for Demographic Research, Rostock, Germany; 2 Jagiellonian University, Institute of Environmental Sciences, Kraków, Poland; University of Innsbruck, AUSTRIA

## Abstract

The role of extrinsic mortality in shaping life histories is poorly understood. However, substantial evidence suggests that extrinsic mortality interacts with density-dependence in crucial ways. We develop a model combining Evolutionarily Stable Strategies with a projection matrix that allows resource allocation to growth, tissue repairs, and reproduction. Our model examines three cases, with density-dependence acting on: (i) mortality, (ii) fecundity, and (iii) production rate. We demonstrate that density-independent extrinsic mortality influences the rate of aging, age at maturity, growth rate, and adult size provided that density-dependence acts on fertility or juvenile mortality. However, density-independent extrinsic mortality has no effect on these life history traits when density-dependence acts on survival. We show that extrinsic mortality interacts with density-dependence via a compensation mechanism: the higher the extrinsic mortality the lower the strength of density-dependence. However, this compensation fully offsets the effect of extrinsic mortality only if density-dependence acts on survival independently of age. Both the age-pattern and the type of density-dependence are crucial for shaping life history traits.

## 1. Introduction

Extrinsic mortality is risk of death that is independent of an organism’s strategy of survival and reproduction [1]. As such, it cannot be avoided. While extrinsic mortality is used in many models and invoked as a key factor affecting life history patterns in many organisms, its role in nature is poorly understood. Some see it as a central driver of life history evolution, while others think it may play no role at all. Opinions on the matter are, of course, influenced by the organism being studied or the assumptions in the model being developed. Here, we show that extrinsic mortality is indeed an important and general force shaping the life histories of organisms, and must certainly be common in nature. We show that the reason its role is controversial is because it interacts with density-dependence such that simple unidirectional hypotheses about its role will often not be supported (such as Williams’ hypothesis, see below). We also show that the type of density-dependence, what it specifically acts on, has significant implications for the effect of extrinsic mortality on the life history.

Part of the confusion about extrinsic mortality stems from its influence on selection pressure. For instance, the observation that selection pressure declines with age after maturity is central to classic theories of ageing. Because mortality limits survival to future ages, later ages contribute less to fitness. Haldane [2] noticed this in 1941, and the idea was furthered by Medawar [3,4], and then Williams [5]. The idea was given needed mathematical form by Hamilton [6]. Declining selection pressure with age is the basis for each of the three main theories of ageing:

1. mutation accumulation theory (MA), where senescence is the unavoidable result of late-acting deleterious mutations that accumulate in a germline [4]);
2. antagonistic pleiotropy theory (AP), where senescence results from a balance between benefits of mutations at early ages and costs at later ages [5,7]; and
3. disposable soma theory (DS), where senescence results from tradeoffs between the allocation of resources to reproduction and somatic maintenance [8].

Because resource allocation must be at least partially dependent on genes, the DS model can be considered a physiological extension of the AP model [9]. Here we directly invoke DS, and thus AP indirectly. For the role of extrinsic mortality on life history evolution under MA see [10], [11] and [12].

Declining selection pressure with age is also related to an influential hypothesis about the effect of extrinsic mortality on senescence. Williams [5] hypothesized that: “low adult death rates should be associated with low rates of senescence, and high adult death rates with high rates of senescence.” Abrams [10,13], Charlesworth [14] and Caswell [15] pointed out that even though Williams' hypothesis is widely quoted as specifically linking extrinsic mortality to senescence, this is not supported by Hamilton’s selection gradients. To understand why this is, one must see that for density-independent populations the appropriate measure of fitness is the intrinsic population growth rate *r* of the Euler-Lotka equation (e.g. [6,14]). Any increase in age-independent mortality is compensated by a decrease in *r*, such that there is no effect of mortality on the age distribution and thus on the force of selection by age. This point has been reiterated many times [10,14–17]. Likewise, Williams’ hypothesis has inspired a great deal of debate and empirical study (e.g. [10–12,15,18–22]), but the results supporting it are mixed, which is not a surprise when one realizes that it is in fact not predicted by the formal theory initiated by Hamilton and modified by many others [10–12,15,23–26].

While many researchers have found the correlations between adult death rate and senescence or lifespan that seem consistent with Williams’ hypothesis [22,27–37], other studies have not [20,21,38,39]. This inconsistent empirical support for Williams’ hypothesis implies that there may be some ecological factors that mediate the effect of declining selection pressure and changes in age-specific mortality. Density-dependence is a strong candidate for such a factor because it can cause feedbacks between mortality pressure and per-capita resource availability [16,20,39]. Populations growing in constant environments must inevitably face some density-dependent effects [23] from factors such as food scarcity or lack of nesting sites (among others).

Abrams [10] provided the seminal work on the effects of extrinsic mortality in various density-dependent (DD) contexts. Abrams distinguished different types of DD, both age-dependent and age-independent. He also demonstrated how the products of *l*_*x*_ (survivorship) and *m*_*x*_ (fertility) can change in response to density and extrinsic mortality. This was an important result, as it showed that the effect of density can be different, depending on assumptions of its age pattern and what it acts upon (fertility or mortality). The genius of Abrams’ model is its simplicity and generality. However, we suggest that extending Abrams’ model to include further ecological realism can help resolve some of the existing tension between theory and data in the study of the evolution of senescence and life history traits in general. To extend Abrams’ results, we explicitly consider tradeoffs predicted by the disposable soma theory of aging. We demonstrate that extrinsic mortality accompanied by DD may shape the timing of maturity (age at first reproduction) and other resource-allocation-related traits. Thus, extrinsic mortality and DD can affect *l*_*x*_ and *m*_*x*_ in different ways depending on trade-offs between them. These tradeoffs are in turn influenced by the allocation patterns to growth, reproduction, and somatic maintenance.

Caswell [15] revisited some of Abrams [10] results and pointed out that Williams’ hypothesis has been frequently misconstrued as a prediction that increased extrinsic mortality should lead to faster ageing. Simply raising (lowering) age-independent extrinsic mortality does not unambiguously predict an increase (decrease) in the rate of ageing. While this point is important, Caswell’s comment had the unfortunate byproduct of amplifying the (incorrect) view that age-independent extrinsic mortality plays no predictive role in life history evolution. His critique was aimed specifically at a common tendency to misinterpret Williams’ hypothesis in light of Hamilton’s indications for the force of selection. Caswell showed that the selection gradient for any life history trait is unaffected by extra mortality, provided there is no age pattern to either the added mortality or to DD. However, this result presumably rests on the assumption that DD acts on mortality in a uniquely age-independent manner and does not consider other types of DD, such as those we consider below. On the other hand, Abrams pointed out that the type of DD (acting on fertility or mortality) is important for predicting the influence of extrinsic mortality on senescence. Across these findings by Abrams and Caswell, the mixed empirical support for Williams’ hypothesis, and a need to extend the ecological realism of evolutionary models, it is clear that the role of extrinsic mortality on life history evolution requires further investigation.

We examine the effect of extrinsic mortality on life histories under different types of DD. We do this using a simple model of resource allocation, which assumes that available resources can be allocated to growth, reproduction, or tissue maintenance (repair). This approach enables studying tradeoffs between age at maturity, body size, and aging, which is not possible using purely demographic models. To model the life history as an evolutionarily stable strategy (ESS) we find the invasion-resistant condition, an approach dubbed the “master fitness” concept by Metz and others [40–42]. The advantage of this approach is that it does not rely on commonly-used fitness measures such as *r*, *R*_0_, or total population size, each of which have shortcomings for answering complex questions about how density-dependence interacts with intrinsic mortality.

## 2. The model

### 2.1. The resource allocation model

In resource allocation models, organisms have an energy budget that must be divided among growth, reproduction, storage, maintenance, or repairs (reviewed in [24,25]). It is assumed that the amount of this resource available per unit of time is determined by body size and the environment. The decision about the share of resources allocated toward each competing end must be a target for natural selection. Earlier life history models based on resource allocation show that under most circumstances resources are optimally allocated to either growth or reproduction and not to these two processes simultaneously, although indeterminate growth can be selected for in a seasonal environment [25,43]. For simplicity, we consider animals with determinate growth, in that the resources remaining after maintenance and repair are allocated to growth until adulthood and to reproduction from maturation onward. We assume a constant fraction of resources allocated to somatic repair throughout the entire lifespan. With these two simplifying assumptions, there are only two traits being directly modeled as targets of natural selection: (i) age at maturity and (ii) the fraction of resources allocated to repairs.

**Fig 1:**
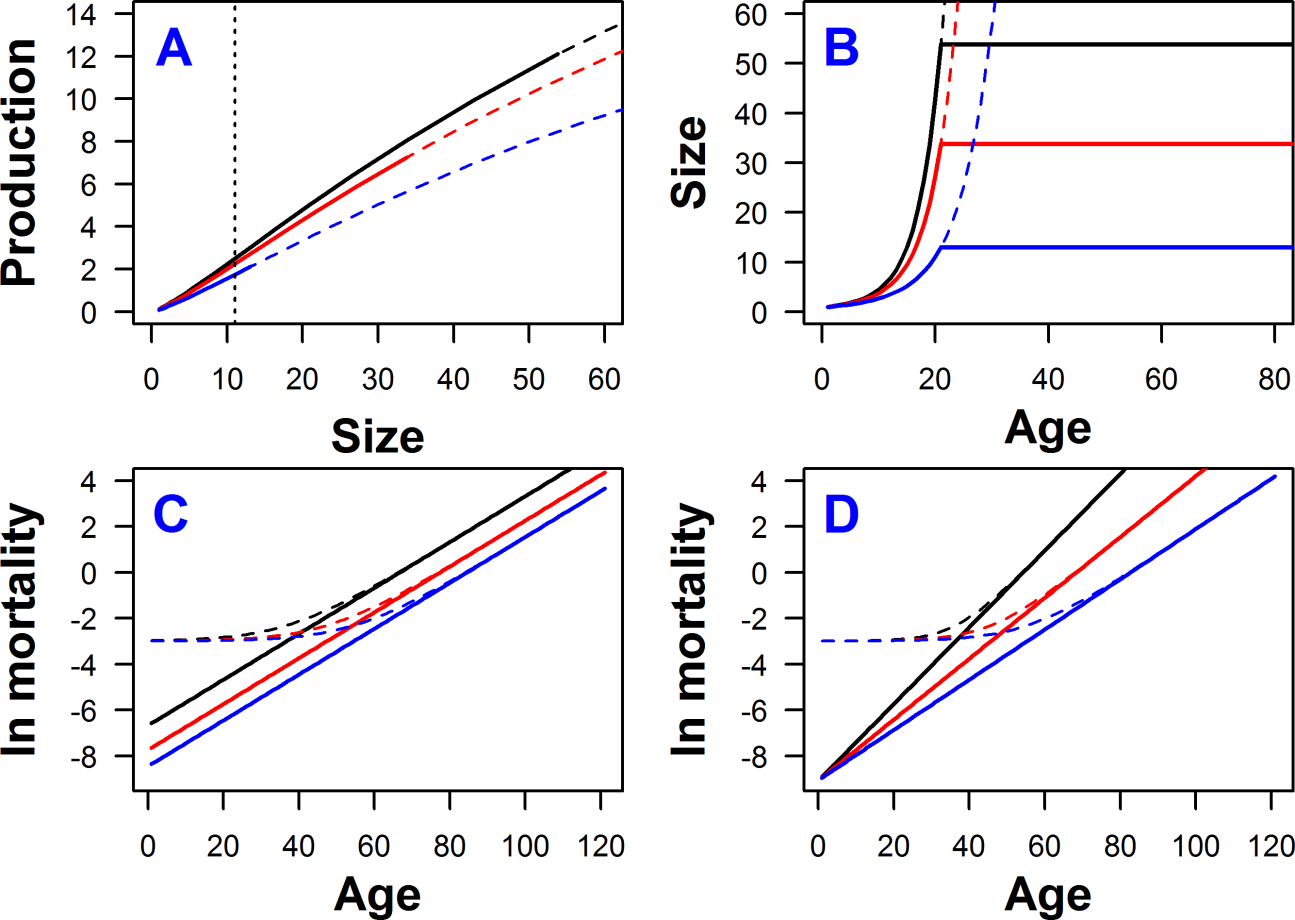
Model assumptions. (A) Production rate as a function of body size for three different age-independent effects of DD on production rate: black lines–no effect of DD (*Q*_*ψ*_ = 1), red lines–moderate DD (*Q*_*ψ*_ = 0.9), blue lines–strong DD (*Q*_*ψ*_ = 0.7). Dashed lines represent the production rate expected if an organism would continue to grow rather than mature. Solid lines represent a part of the production curve that is experienced by an organism maturing at age 20. The ends of the solid lines determine adult size and thus fertility rate. The vertical dotted black line denotes the body size at the inflection point, which is common for all three curves. (B) Growth trajectory as a function of age. Colors have the same meaning as in (A). Dashed lines represent the projection of growth trajectories if maturity would not occur. The panels (A) and (B) can also represent the case when there is no DD acting on production rate and different colors indicate different fractions of resources allocated to repairs; black: *z* = 0, red: *z* = 0.1, blue: *z* = 0.3. (C) Mortality as a function of age under the proportional hazard model (PHM); black lines: fraction of resources allocated to repairs *z* = 0.1; red lines: *z* = 0.2, blue lines: *z* = 0.3; solid lines: no extrinsic mortality *c* = 0, dashed lines: *c* = 0.05; other parameters in Eq (5) were set to *a*_1_ *= –*1, *b*_1_ = 0.1, and *u*_1_ = –10. (D) The same as in (C), but under senescence slowing model (SSM); parameters of Eq (6) set to *a*_2_ = –9, *b*_2_ = 0.35, and *u*_2_ *=* 0.325.

The resources available after metabolic costs are subtracted are defined by a production rate function *ψ*. Traditionally, this relationship is approximated by an allometric function of body size *W*, with exponent less than one and probably near 0.7 [31]. Allometric production functions are sufficient for many applications, but do not allow for an inflection point, a plateau, or a decrease of production rate at very large size [44,45]. We use a function for the size-dependence of production rate that allows for an inflection point and a plateau. This “generalized allometric function” reduces to the standard allometric function when the exponent *γ* is set to zero:

$$\psi (x,W)=Q_{\psi }\frac{\alpha W^{\beta }}{{\left(W+\kappa \right)}^{\gamma }},$$ (1)

where body size *W* is a function of age *x*. The function *Q*_*ψ*_ (Eq (11)) models the effect of density on production rate, which for different *x* can take values from the range [0,1]. When *Q*_*ψ*_ = 1 for every *x* density has no effect at any age. The parameter values in (1) have no qualitative effects on the results, provided they are kept in reasonable ranges resulting in non-decreasing population growth at ESS and giving strategies for which the probability of surviving beyond the last age-class is negligible. Fig 1A illustrates an example of function (1) with *α* = 45, *β* = *γ* = 1.25 and *κ* = 100; If *γ* = 0 the function loses an inflection point, which for some types of density dependence (e.g., DD acting uniformly on survival) may lead to unrealistic results (such as maturity in the first age class).

The units for resource/energy/size are normalized by assuming that organisms start growth at size *W*_0_ = 1. As such, resources, energy, and size have the common unit equal to the energy content of a newborn individual. From the size of independent growth *W*_0_ = 1 until maturity *τ* individuals grow according to the equation:

$$\frac{dW}{dx}=\left(1-z\right)\psi (W,x)$$ (2)

where *z* is the fraction of resources allocated to repairs. This equation is solved numerically (Runge-Kutta methods, *deSolve* R-package [46]) to obtain size at maturity *W*_*τ*_. Example growth trajectories are presented in Fig 1B.

After reaching age *τ* and corresponding mature size *W*_*τ*_, available resources are allocated to reproduction and converted to offspring. Density-dependence may act on fertility *m*_*x*_, which is modeled by the function *Q*_*R*_ (see Eq (11) for details). The fertility for ages from maturity onward is given by

$$m_{x}=Q_{R}(1-z)\psi (W_{\tau },x)$$ (3)

If DD does not act on fertility, then *Q*_*R*_ = 1 for all *x*. If age at maturity *τ* is a fraction of a particular age class *x* then interpolation is used (see S1 File).

Ageing is typically considered as a process resulting from the balance between accumulation of damage and the fraction of resources used for maintenance and repairs [8,18,47,48]. We did not consider accumulation of damage explicitly, but we used models based on the empirically driven assumption that intrinsic mortality increases faster than linearly with age [49]. In general, to model density-independent age-specific mortality we used the Gompertz-Makeham function [50,51]:

$$\mu_{x}=\exp(a+bx)+c$$ (4)

where exp(*a*) is defined as initial mortality, *b* is the rate of aging and *c* is age-independent extrinsic mortality (the results shown assume *c* equal to 0, 0.025 or 0.05). We consider two models, both adopted from survival analysis, that can be used to convert allocation to repairs *z* into units of mortality. In each model, allocation to repairs can affect the scale or the shape of log-intrinsic mortality. The first is based on a population proportional-hazard model (PHM, e.g. [52]) in which a covariate proportionally affects the mortality rate. In our model, allocations to repairs affect initial mortality exp(*a*), and leave the rate of aging unaffected. The fraction of resources allocated to repairs *z* is transformed into mortality units via the function *φ*(*z*,*u*,*K*) (Eq (7)), where *K* is repair efficiency, taking values 3, 4 or 5 (results for other values of *K* are presented in S2 File). The PHM formula for mortality used here is:

$$\mu_{x}=\exp(a_{1}-\varphi (z,u_{1},K)+b_{1}x)+c$$ (5)

where *a*_1_ *= –*1, the rate of aging *b*_1_ takes values 0.075, 0.1, or 0.125, and *u*_1_ = 10 is a constant (see Eq (7)). The PHM model is depicted in Fig 1C. The assumed values for *a*_1_ and *u*_1_ give a wide range of possible repair-dependent initial mortalities that lie between exp(–11) and exp(–1) for different *z*. For relatively high values of *u*_1_, there is a strong effect of repairs on intrinsic mortality.

In the second model for converting repairs into mortality, allocations affect the rate of aging (senescence-slowing model, SSM, see [53] for analogical population model). In this case the rate of aging is the difference between a constant *b*_2_ = 0.35 and *φ*:

$$\mu_{x}=\exp(a_{2}+(b_{2}-\varphi (z,u_{2},K))x)+c$$ (6)

where *a*_2_ takes values –8, –9, or –10, and *u*_2_ = 0.325 is a constant (see Eq (7)). The SSM model is illustrated in Fig 1D. These values for *b*_2_ and *u*_2_ give a wide range of possible repair-dependent aging rates for different *z* that are all between 0.0025 and 0.35. As in the PHM, relatively high values of *u*_2_ indicate a strong relationship between repairs and intrinsic mortality.

For both PHM and SSM models, the function *φ*(*z*,*u*,*K*) takes the form:

$$\varphi (z,u,K)=uz^{\frac{1}{K}}$$ (7)

where *u* is arbitrarily chosen to scale the effect of repairs on mortality. Results of the model are qualitatively similar if *u*>0 (*u* = 0 means no effect of repairs on mortality). We chose values for *u* that ensure mortality remains reasonably high (non-negligible) across age classes.

We use the model with discrete age classes. The probability of survival for each age class is the product of the DD factor acting on survival *Q*_*S*_ (see Eq (11) for details) and the density-independent probability of survival of an age class *x*. The conversion of the force of mortality *μ* to the probability of survival of an age class is based on a commonly used approximation (midpoint rule):

$$p_{x}\approx Q_{S}(N,x)\exp\left(-\mu_{x+0.5}\right)$$ (8)

Survivorship *l*_*x*_, the probability of surviving from birth to age *x*, is defined as the product of consecutive probabilities of survival:

$$l_{x}=\prod_{y=0}^{x-1}p_{y}$$ (9)

with *l*_*0*_ = 1

### 2.2. Modeling ESS in a density-dependent population

As a building block of our model we use the birth-pulse and post-breeding [54,55] projection matrix

$$A=\left[\begin{array}{cccccc} f_{0} & f_{1} & f_{2} & \cdots & f_{\omega -2} & f_{\omega -1}\\ p_{0} & 0 & 0 & \cdots & 0 & 0\\ 0 & p_{1} & 0 & \cdots & 0 & 0\\ 0 & 0 & p_{2} & \cdots & 0 & 0\\ \vdots & \vdots & \vdots & \ddots & \vdots & \vdots \\ 0 & 0 & 0 & \cdots & p_{\omega -2} & 0 \end{array}\right]$$ (10)

where *ω* is the largest age class, *p*_*x*_ is probability of survival of age class *x*, *m*_*x*_ is mean fertility in age class *x*, and *f*_*x*_ = *p*_*x*_*m*_*x*+1_ is effective fecundity in age class *x*. The projection matrix characterizes population growth according to the formula: **N**_*t*+1_ = **AN**_*t*_, where **N**_*t*_ is a vector with elements *n*_*x*,*t*_ describing population number at age *x* and time *t*. The intrinsic growth rate *r* of a population is the natural logarithm of the dominant eigenvalue of **A**. The parameters of **A** are dependent on a strategy defined by age at maturity *τ* and the fraction of resources devoted to repair *z*. Density-dependent populations can reach an equilibrium **Ñ,** but may also fluctuate cyclically or even chaotically [55,56]. For the range of parameters considered here, stable equilibria were always reached and were found numerically.

DD is introduced into the model by making some of the elements of **A** dependent on population size *N*. We assume that individuals affect DD the same way regardless of age class [55,57–59]. We also assume that each strategy is equally affected by DD. These simplifying assumptions are necessary because considering impacts that vary with age-class would produce an unmanageable number of sub-models that would distract from the focus of the analysis. We consider three general types of DD, with density acting directly on: (i) fertility, (ii) survival, and (iii) production, denoted by *Q*_*R*,_ *Q*_*S*_, and *Q*_*ψ*_, respectively. If population size is regulated by the mortality or out-migration of juveniles, it is mathematically equivalent to DD acting on fertility [10,14]. Density-dependence may act equally (uniformly) on all age classes or be age-specific. We model DD according to a Beverton-Holt function (e.g. [55,59]), with power vector *B*(*x*) added to capture the age-dependence of the density effect (see S3 File for more details):

$$Q(N,x)={\left(\frac{1}{1+vN}\right)}^{B(x)}$$ (11)

where *N* equals the sum of each element of **N**_*t*_. The vector *B*(*x*) equals 1 for all age classes if they are equally affected by density. The parameter *v*, arbitrarily set to 10^−4^, determines the strength of DD, and also affects carrying capacity.

The main goal of this paper is to find ESS strategies, defined here as evolutionarily stable allocation strategies (combination of age at maturity and fraction of resources allocated to repair) under different types of DD and specifically to examine the impact of age-independent extrinsic mortality on such unbeatable strategies. We find the ESS by using the invasion criteria of [40]. This means that the ESS strategy cannot be invaded by any other strategy. That is, at the invasion the resident phenotype *θ*_*R*_ is assumed to be at population size and age-structure equilibrium *N*_*R*_ as described by the matrix **A**[*θ*_*R*_, *N*_*R*_]. Then this resident phenotype is invaded by another strategy *θ*_*I*_. Because the invading phenotype is initially small in number, its dynamic approximately depends on the population size of the resident phenotype, given by the matrix **A**[*θ*_*I*_, *N*_*R*_]. If an invader can increase in number then the invasion is successful, otherwise it fails. If the invading strategy wins then it becomes the new resident strategy. Trying all allowable strategies, the ESS strategy θ^*^ = {τ*, z*} is established for a given set of model parameters. More formal description of the algorithm used for seeking ESS is given in S4 File. For further details about modeling ESS in density-dependent models see [40–42,55,59–61].

## 3. Results

### 3.1. Density-dependence acting uniformly on all age classes

**Fig 2:**
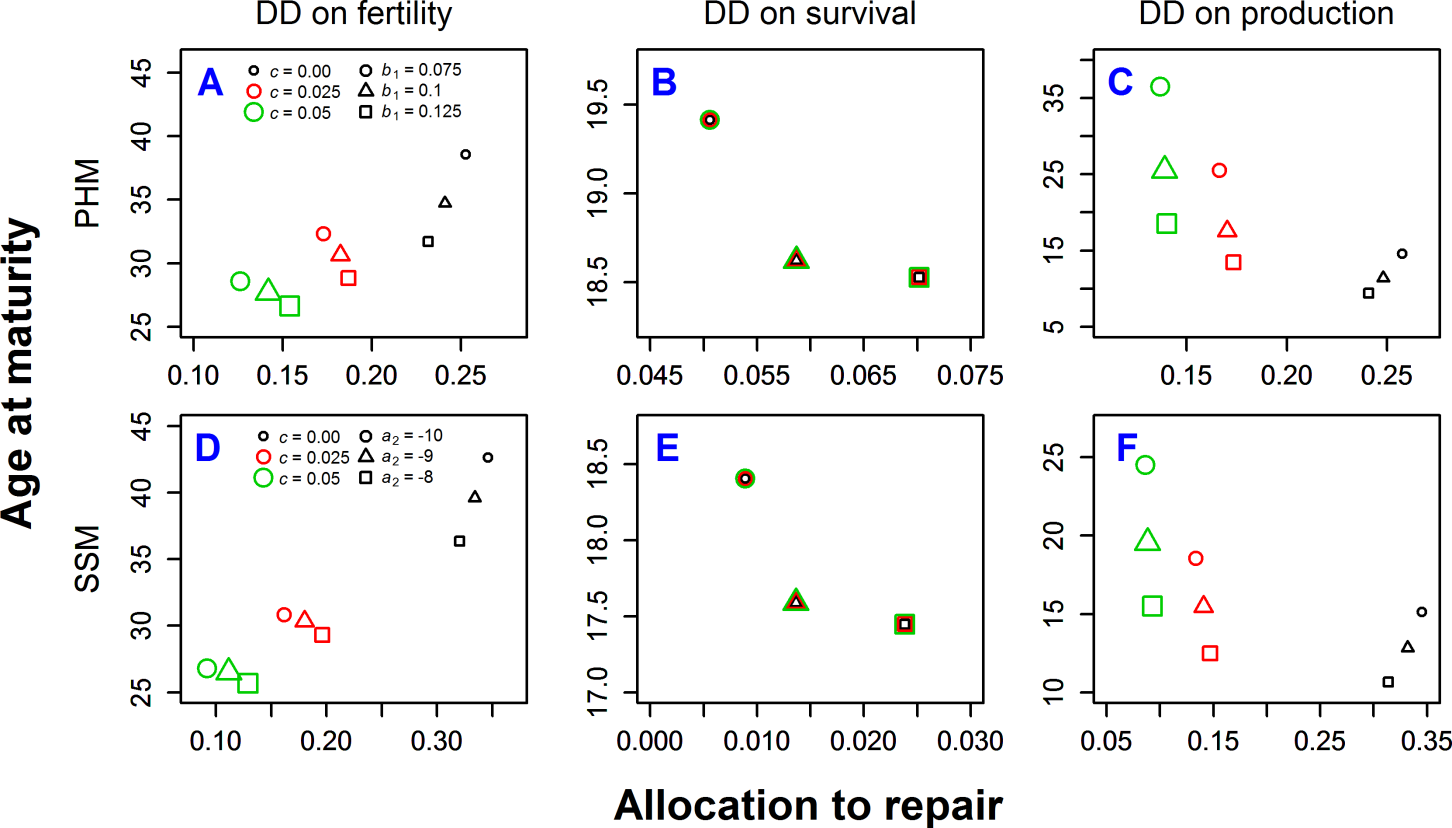
ESS age at maturity and allocation to repair for different types of density-dependence (columns) and the different effects of repair on Gompertz-Makeham parameters (rows). Different levels of extrinsic mortality *c* are denoted by size and color of the markers and different mortality parameters *b*_1_ (for PHM) or *a*_2_ (for SSM) are denoted by shapes of markers. All cases of DD are age-independent. The same repair efficiency is assumed for all panels (*K* = 4). See S2 File for results under other repair efficiencies.

Across all scenarios examined, we find that each type of DD has different effects on the ESS life history. We also find that results are often qualitatively similar across both models linking repairs to mortality (PHM and SSM). For instance, when DD acts uniformly on fertility, the results that increased extrinsic mortality *c* leads to decreases in the age at maturity and the fraction of resources allocated to repairs holds across all sets of parameter values analyzed (Fig 2A and 2D; see also Fig 1AD in S2 File for the effect of repair efficiency). However, when DD acts uniformly on mortality, extrinsic mortality has no effect on the ESS (Fig 2B and 2E and Fig 1BE in S2 File; points for different *c* are superimposed). When DD acts uniformly on production rate, increasing extrinsic mortality *c* leads to lower allocations to repairs, but later maturity (Fig 2C and 2F and Fig 1CF in S2 File).

In addition to the role of age-independent extrinsic mortality, we also investigated the effect of the rate of aging *b*_1_ in PHM, and the intercept of the log of intrinsic mortality *a*_2_ in SSM on ESS (Fig 2). Under PHM, maturity occurs earlier under high values of *b*_1_, indicating a steep increase of mortality with age (Fig 2A, 2B and 2C). However, the effect of the rate of aging *b*_1_ on allocation to repairs varies between different types of DD. The allocation to repairs clearly interacts with extrinsic mortality *c*, except for the case when DD acts uniformly on survival (Fig 2B), where extrinsic mortality has no impact on the ESS allocation. For DD acting uniformly on fertility or production, allocations to repair decrease with increasing rate of aging *b*_1_ when there is no extrinsic mortality (*c* = 0) and increase with *b*_1_ when extrinsic mortality is present (*c* equal to 0.025 or 0.05) (Fig 2A and 2C). Under SSM *a*_2_ has qualitatively the same effect on allocation strategies as *b*_1_ in PHM (Fig 2D, 2E and 2F).

The strength of DD, measured as (1–*Q*), correlates negatively with age-independent extrinsic mortality. The higher the extrinsic mortality, the lower the strength of DD required to maintain the population at equilibrium. The mechanism for this, called compensation, is discussed below.

**Fig 3:**
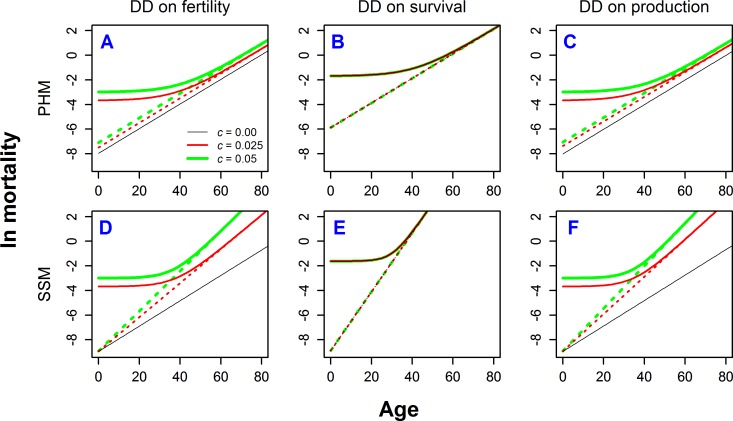
Mortality rates for ESS under different types of density-dependence. Each case corresponds with those represented by triangles in Fig 2. Solid lines represent total mortality and dashed lines intrinsic mortality. Different colors and line widths represent different levels of extrinsic mortality *c*.

In Fig 3 we show six mortality patterns that correspond to the cases denoted by triangles in Fig 2. Under PHM, only the intercept of intrinsic mortality, marked with dashed lines, can respond to change in *c* through its influence on repair strategies. As such, the slope of the natural logarithm of intrinsic mortality (rate of aging) is fixed (see model description). Conversely, in the SSM model the intercept is fixed, but the slope of the natural logarithm of intrinsic mortality responds to *c*. For DD acting on fertility (Fig 3A and 3D) or on production rate (Fig 3C and 3F) we observe qualitatively similar results. The higher the extrinsic mortality, the higher the intercept of intrinsic mortality for PHM or the higher its slope for SSM. Consequently, overall mortality is higher in both cases for high *c* (solid lines, Fig 3). When DD acts uniformly on survival (Fig 3B and 3E), extrinsic mortality has no effect on intrinsic mortality or overall mortality. This is because overall mortality combines three kinds of mortality: intrinsic (dependent on allocation strategy as modeled by PHM or SSM), extrinsic (age-independent), and that coming from DD (with the strength dependent both on intrinsic and extrinsic mortality). For this kind of DD full compensation of extrinsic mortality by density-dependent mortality occurs, meaning that an increase in mortality rate by a certain unit will be completely offset by a decrease of mortality resulting from DD.

**Fig 4:**
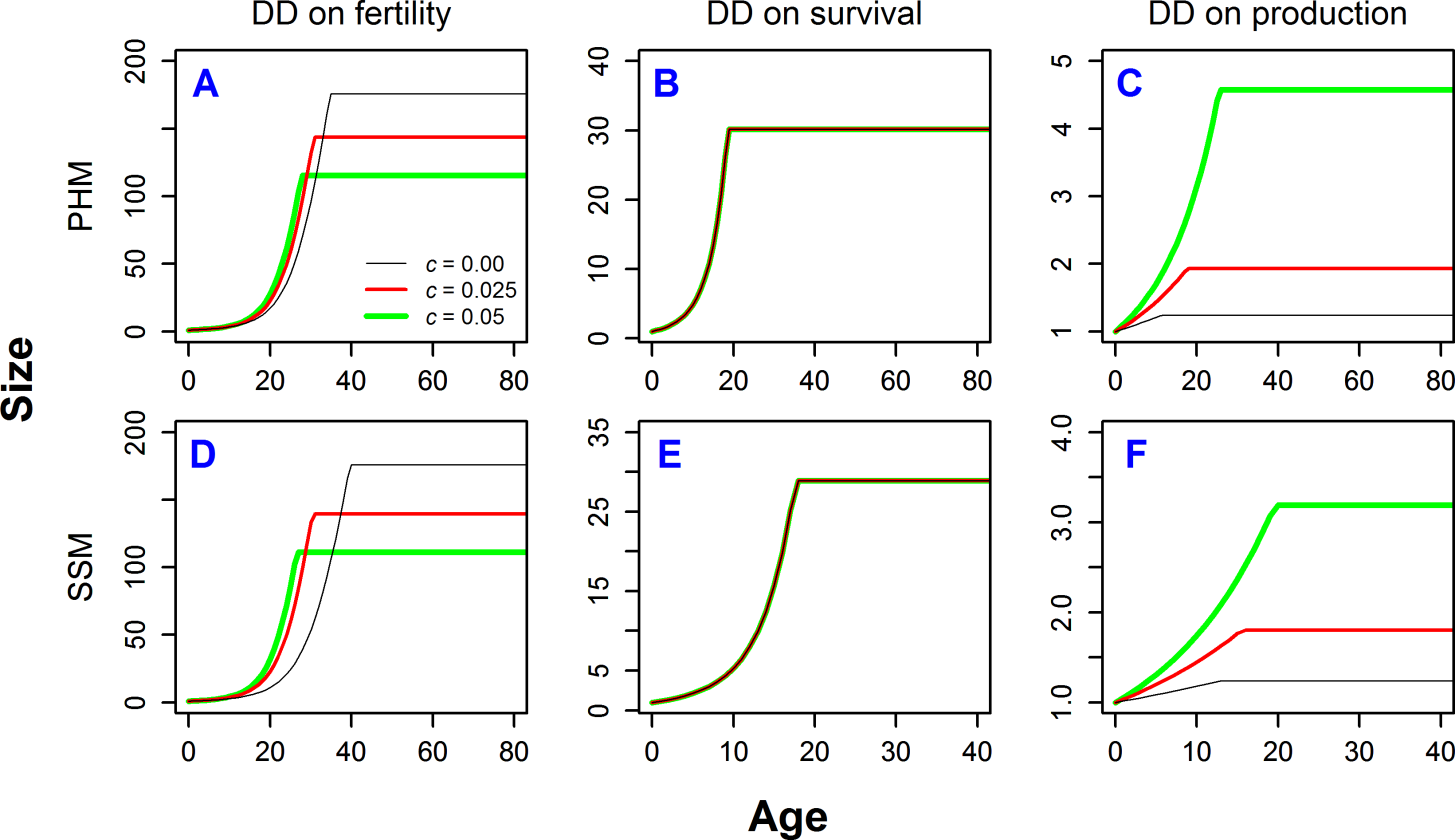
Growth trajectories for ESS under different types of density-dependence. Growth trajectories correspond to the cases shown by triangles in Fig 2. Different colors and line widths represent different levels of extrinsic mortality *c*.

Fig 4 shows ESS growth trajectories for cases depicted in Figs 2 and 3. Two models for the dependence of mortality on repair (PHM and SSM) give qualitatively similar results. When DD acts uniformly on fertility (Fig 4A and 4D) then higher extrinsic mortality induces faster growth, but maturity is reached earlier and at smaller sizes. When DD acts uniformly on survival (Fig 4B and 4E), extrinsic mortality has no impact on growth trajectories. When DD acts uniformly on production rate (Fig 4C and 4F) then higher extrinsic mortality leads to faster growth, later maturity, and larger adult size. One should notice that DD acting on fertility and DD acting on production rate have essentially the opposite consequences for age and size at maturity.

### 3.2. Density-dependence varying with age

**Fig 5:**
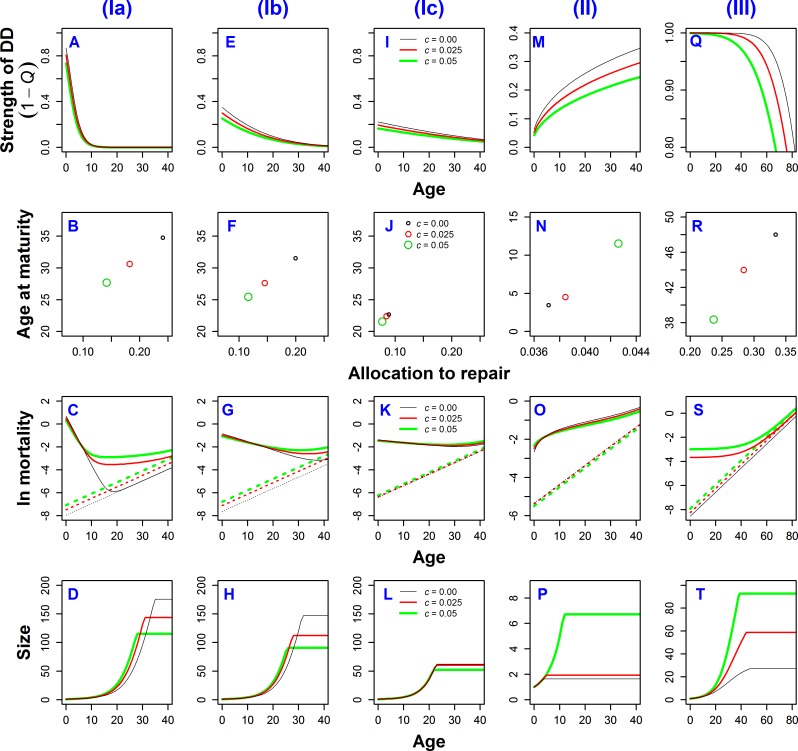
ESS life histories for three different cases of age-specific density-dependence. (**I**) DD acting on survival with strength decreasing with age, (**II**) DD acting on survival with strength increasing with age, (**III**) DD acting on production rate with strength decreasing with age. All cases calculated for PHM, with parameters *b*_1_ = 0.1, and *K* = 4. Strength of DD was calculated as 1–*Q*, where *Q* (*Q*_*S*_ or *Q*_*ψ*_) is defined in Eq (11). See also S3 File for mathematical definitions and parameters for used shapes of DD.

So far, we have considered only age-independent DD. To illustrate the effects of age-varying DD we consider three cases as the most interesting and biologically relevant (Fig 5). These examples demonstrate that the pattern of DD is pivotal for determining the role of extrinsic mortality on different life-histories. The three cases we examine are: (**I**) DD acts on survival with strength that decreases with age; (**II**) DD acts on survival with strength increasing with age; and (**III**) DD acts on production rate with strength that declines with age.

For case (**I**), DD acts on survival with strength that decreases with age (represented as 1–*Q*, see Eq (11)) (Fig 5A), which is probably the most frequent type of DD found in nature (see discussion). In instances (**Ia**), (**Ib**), and (**Ic**), we vary the rate at which the strength of DD decreases with age. In case (**Ia**), the effect of extrinsic mortality *c* on age at maturity and growth rate is essentially the same as under DD acting uniformly on fertility (compare Fig 5B with triangles in Fig 2A, and Fig 5D with Fig 4A). This is because the effect of density on fertility is mathematically identical to the effect of density on juvenile mortality or migration. The kind of DD in (**Ia**) also gives the same intrinsic mortality patterns (compare Fig 5C with Fig 3A, dashed lines), but the effect of extrinsic mortality on overall mortality is obviously different (solid lines). In cases (**Ib**) and (**Ic**), where the strength of DD declines at slower rates, the effect of extrinsic mortality on life history allocation strategies also decreases, approaching the case when DD acts uniformly on survival (Figs 2B, 3B and 4B). This is because the effect of mortality is enhanced by DD acting on juveniles, but diminished by DD on adults.

In case (**II**) the strength of DD acting on survival increases with age (Fig 5M). Higher extrinsic mortality leads to later maturity and a greater fraction of resources allocated to repairs (Fig 5N), which in turn results in lower intrinsic mortality (Fig 5O dashed lines). Furthermore, overall mortality at early ages is higher for high c, but the order reverses at later ages. Such a shift is opposite that of case (**I**) (compare the order of different solid lines in Fig 5C and 5O). The later maturity at higher *c* results in longer growth and larger adult sizes, while the increased allocations to repairs result in slightly slower growth (Fig 5P).

In case (**III**) DD acts on production rate with strength that decreases with age (Fig 5Q). This occurs in nature when DD acts mainly on growing animals. The results are qualitatively similar to DD acting uniformly on fertility in terms of the effect of extrinsic mortality on ESS resource allocation (Fig 5R) and underlying mortality patterns (Fig 5S). However, the key difference is how the growth rates are affected: higher extrinsic mortality leads to faster growth (as in the case of DD acting uniformly on fertility), but it also increases the ESS size at maturity (compare Fig 5T with Fig 4A).

## 4. Discussion

The role of density-dependent effects has been under-studied in life history theory, in spite of the fact that most populations experience DD in some way [62–66]. In this paper we investigate how the effect of age-independent extrinsic mortality shapes life history traits, such as age and size at maturity, growth rate, allocation of resources devoted to repairs, and patterns of senescence, under various types of DD. We have chosen three illustrative examples, the consequences of DD acting on: survival, fertility, and production rate. Production rate, in particular, has not been given much attention by life history theorists (but see [67] for a simple model of density acting on juvenile growth). It is also under-studied empirically (but see [68] for an example of density-dependent metabolic rate in protists).

### 4.1. William’s hypothesis

We added further confirmation to the fact that Williams hypothesis [5] is not a general biological principle [10,14,15,69]. While high extrinsic mortality can lead to faster senescence in some density-dependent scenarios, like when DD acts on fertility or juvenile survival, there are also cases in which extrinsic mortality is irrelevant to aging (when DD acts uniformly on survival) or even delays aging (when DD acts most strongly on old individuals, see also [10,70]).

Perhaps the best-known empirical exception to Williams hypothesis, where the opposite of the prediction was observed empirically, are Reznick et al.’s experiments on senescence in guppies [20]. They found that guppies from high predation environments had much longer lifespans and reproductive lifespans than those from safer habitats. Several explanations have been proposed for this finding (reviewed in [13]), one of which is that DD was acting more strongly on the survival of older individuals. We reanalyzed this case using our resource allocation model (Fig 5 case (**II**)) and found that when the strength of DD increases with age, higher extrinsic mortality indeed reduces the rate of senescence, but also leads to larger size at maturity via delayed maturation. However, we must also point out that the effect of extrinsic mortality on senescence is rather minor (Fig 5O). This probably results from the simplifying assumption that the fraction of resources allocated to repair is fixed across the lifespan, which could be explored further. Overall, different types of DD give different results for the influence of extrinsic mortality on the rate of senescence.

### 4.2. The role of age-independent extrinsic mortality cannot be neglected

Age-independent extrinsic mortality is frequently ignored. For example, Caswell [15] noted that selection gradients of invasion exponents should be unaffected by extrinsic mortality: “If neither the extrinsic mortality nor the density-dependence are age specific, then the extra mortality has no effect on the pattern of selection gradients.” This observation should be restricted to the case of DD acting uniformly on survival. In a density-dependent system not only the population growth rate *r*, but also the strength of DD and equilibrium population density respond to additional extrinsic mortality. Compensation, as described below, can offset the effects of extrinsic mortality only where DD acts uniformly on survival. Density-dependence that acts uniformly on fertility will affect the eigenvectors, underlying age structure, and ESS life history. This results in extrinsic mortality having a strong impact on maturation and allocation to repairs, and thus on body size and longevity as well. As mentioned before, DD acting on juvenile mortality/migration is mathematically equivalent to DD acting uniformly on fertility. Given the results presented here, extrinsic mortality should be a common factor shaping life histories in nature. Likewise, debate over the role of extrinsic mortality has been influenced by the structure of the models used to study it; namely, the type of DD assumed and how the age/stage effects of DD are included. Our results suggest that extrinsic mortality will often have an effect on life history evolution.

Ricklefs [71] analyzed connections between rates of aging and different life history parameters. His general conclusion was that aging is most closely correlated with age at sexual maturity through the underlying correlation of these two variables with extrinsic mortality. These conclusions support the hypothesis that DD acting on juveniles prevails in nature (see also [31,44]). Furthermore, Ricklefs also suggests that density-dependent competition with older, socially-dominant, individuals is behind these results. This is similar to our result that strong DD on juveniles tends to increase the effect of extrinsic-mortality on the ESS life history.

### 4.3. Extrinsic mortality shapes senescence, and other life history traits

The most influential work about the types of DD has focused on the effect of extrinsic mortality on senescence [10,14]. By using the resource allocation approach, we extend this earlier work by also investigating the effect of extrinsic mortality on other life-history traits including (i) growth rate, (ii) age, and (iii) size at maturity. Extrinsic mortality affected all of these traits. The only exception occurs when DD acts on survival uniformly with age (Fig 2B and 2E and Fig 4B and 4E). Growth rate is by definition directly connected with allocation to repairs: if more resources are devoted to repair, fewer are left for growth [18,72]. As a result, the instances in our model that seem consistent with the Williams’ hypothesis also have implications for other traits. For example, higher extrinsic mortality leads to a reduced fraction of resources invested in repairs, which in turn leads to faster growth rate (Figs 2, 3 and 4 left and right panels, Fig 5 cases (**I**) and (**III**)), but also to lower adult size because of a shortened period of growth (Figs 2 and 4 left and right panels). When the strength of DD decreases with age, the effect of extrinsic mortality on the ESS depends on the rate of this decrease. If the decrease with age is fast, the effect is strong as in the case of DD acting on fertility or juvenile survival. If the decrease is slow, the effect of extrinsic mortality on the ESS is weak and may nearly disappear, approaching the case with DD acting uniformly on survival (Fig 5 cases (**Ia**), (**Ib**), and (**Ic**)). The reverse effect, although weak, is observed when DD acts on production rate (Figs 2, 3 and 4 right panels, Fig 5 case (**III**)): growth rate accelerates under high extrinsic mortality due to reduced allocations to repairs and reduced DD on production rate in older animals.

Age at maturity is not directly connected with allocation to repairs. For most of the cases of DD considered here, we observed that the higher the mortality the earlier the maturity (Figs 2, 3 and 4 left panels, Fig 5 cases (**I**) and (**III**)), but with notable exceptions (Figs 2, 3 and 4 right panels, Fig 5 case (**II**)). For example, when DD acts on production rate, the ESS under age-independent DD at high extrinsic mortality was later age at maturity (longer growth period). The ESS under DD that decreases with age was younger age at maturity (shorter growth period). Size at maturity is a direct outcome of growth rate and age of maturity. Higher extrinsic mortality leads to smaller size only when DD acts on fertility uniformly with age, or when DD has greater strength on juvenile than adult survival (Figs 4A and 4D and 5D, 5H and 5L). In all other cases, increased extrinsic mortality leads to either no change in size at maturity (Fig 4B and 4E) or larger size (Figs 4C and 4F and 5P and 5T).

### 4.4. Extrinsic mortality interacts with density-dependence via a compensation mechanism

Higher levels of extrinsic mortality require lower levels of DD to maintain a stable population (Fig 5A, 5E, 5I, 5M and 5Q, see also [73,74]). This compensation alters the effect of extrinsic mortality on life history traits. Any increase in age-independent extrinsic mortality leads to a decrease in population size until a new equilibrium is reached. At the new equilibrium, the smaller population also experiences reduced strength of DD, expressed as 1–*Q*, because *Q* is a decreasing function of population size.

When DD acts uniformly on survival (Figs 2, 3 and 4, middle panels) then the effects of extrinsic mortality are completely compensated for by DD, which we call “full compensation.” Full compensation explains why this kind of DD prevents extrinsic mortality from shaping life histories (see also [10,14 p.196]). The effect of DD on the life history depends on its age pattern, especially on how much stronger the effect of DD is experienced by juveniles compared with adults. If DD acts primarily on juveniles (pre-reproductive ages, generally) and is negligible later in life, extrinsic mortality has a strong impact on ESS life histories (Fig 5, case (**Ia**)). This is because a change (increase/decrease) in juvenile mortality is mathematically equivalent to a change in fertility, and compensation via fertility cannot offset changes in extrinsic mortality. When DD affects adult survival then extrinsic mortality has a diminished effect on ESS life histories, because changes in extrinsic mortality can be partially offset (Fig 5, case (**Ib**) and (**Ic**)).

When DD acts uniformly on fertility (Figs 2, 3 and 4, left panels, see also [10,14 p.196]), increased extrinsic mortality leads to fewer individuals, and weaker DD on fertility is required to stabilize the population. Compensation works only on fertility, not survival. The increase in fertility that results from reduced DD has no impact on the evolutionarily stable allocation strategy, because the increase in fertility is proportionally the same for all considered strategies (it does not change the relative fitness of any strategy). Under this DD scenario, extrinsic mortality has a decisive role in shaping life histories leading to earlier maturity, lower allocation to repairs, faster senescence, and faster growth (Fig 2B and 2E). In nature, this scenario seems to hold even for size-dependent extrinsic mortality [73,74].

If DD acts uniformly on production rate (Figs 2, 3 and 4 right panels), the increase in extrinsic mortality also leads to reduced strength of DD. As the direct result of this compensation, we expect faster growth and higher fertility. This also indirectly influences the resulting evolutionarily stable allocation strategies. In principle, higher extrinsic mortality decreases the chance of reaching maturity. This in turn is expected to shorten the growth period. However, we observe the opposite result when DD acts uniformly on production: higher extrinsic mortality leads to later maturation and decreased allocation to repairs (Fig 2C and 2F). This happens because: (i) higher investments in growth are possible due to gains from reduced DD on production rate, (ii) future reproductive gains from higher investments to growth can overcome the loss from increased extrinsic mortality. If DD acts on production rate with strength decreasing with age (Fig 5 case (**III**)), higher extrinsic mortality leads to increased production rate. However, this gain is minimal early in life (Fig 5Q, curves diverge with age) and leads to a stronger effect of extrinsic mortality on pre-reproductive ages. As a result, we observe earlier maturity even if size and growth rate are higher.

## 5. Conclusions

1. DD is often neglected in evolutionary biology. However, it’s importance is clearly indicated by empirical studies (DD is detected in most populations) and theoretical models (it is difficult to understand some evolutionary processes without referring to DD).
2. Williams hypothesis is often incorrectly interpreted as a general prediction, but in fact extrinsic mortality can have different effects depending on the ecology or type of DD acting on the population.
3. Extrinsic mortality plays a crucial role in shaping life history traits in most organisms, often via interaction with DD.
4. Different types of DD cause extrinsic mortality to have different effects on age/size at maturity, growth rate, allocations to repairs, and mortality patterns. Although, there are cases when the effect of extrinsic mortality can be neglected, namely, density-independent population growth or density acting on survival uniformly with age; some evidence suggests that the most frequent regulation of natural populations is density acting on fecundity or juvenile mortality.
5. Compensation is an important mechanism for understanding how DD interacts with extrinsic mortality (higher mortality results in weaker DD). Compensation may be responsible for a great deal of the life history variation observed in the field or the lab. This is because the effect of higher mortality is fully offset with only specific types of DD, such as when DD acts uniformly on mortality rate or when DD does not exist at all.
6. Our results are qualitatively similar for both models converting allocations to repairs into mortality (PHM, where repairs affect mortality proportionally, and SSM, where repairs affect rate of aging). This implies that the qualitative effect of DD on the role of extrinsic mortality in shaping the ESS life history should be taken as independent of the assumed mechanism of aging.

## Supporting information

S1 File Interpolating fractional maturity ages. (PDF)

S2 File Effect of repair efficiency. (PDF)

S3 File Definition of the shapes of density-dependence used in the paper. (PDF)

S4 File Algorithm for searching ESS. (PDF)
