# Supplementary material for: Density-dependence interacts with extrinsic mortality in shaping life histories
Source: PLoS One. 2017 Oct 19;12(10):e0186661. doi: 10.1371/journal.pone.0186661 (PMC5648222; doi:10.1371/journal.pone.0186661)
Supplement: S1 File — (PDF) [file pone.0186661.s001.pdf]

S1. Interpolating fractional maturity ages

Resources allocated to reproduction are the product of density-dependent effects acting on fertility $Q_R(N, x)$ and the production rate at adult size W_τ (size at maturity):

$$m_x = Q_R(N,x)\psi(W_\tau,x)\left[1_{x>\lfloor\tau\rfloor}(x) + (\tau - \lfloor\tau\rfloor)1_{x=\lfloor\tau\rfloor}(x)\right] ,$$

where $1_{t\geq\lfloor\tau\rfloor}(t)$ is an indicator function, $\lfloor\tau\rfloor$ is a floor function, and $(\tau - \lfloor\tau\rfloor)$ is a fractional part of τ. The term in the square bracket performs a simple interpolation when τ is not an integer.
