# Supplementary material for: Density-dependence interacts with extrinsic mortality in shaping life histories
Source: PLoS One. 2017 Oct 19;12(10):e0186661. doi: 10.1371/journal.pone.0186661 (PMC5648222; doi:10.1371/journal.pone.0186661)
Supplement: S2 File — (PDF) [file pone.0186661.s002.pdf]

S2. Effect of repair efficiency

Another important parameter influencing the ESS is repair efficiency K. In most of the cases we consider, increased repair efficiency reduces the fraction of resources invested to repairs (Fig 1A-E in S2). The decrease in allocation to repair that occurs with K is more pronounced in PHM than in SSM. In the case of density-dependence acting on production rate under SSM (Fig 1F in S2) the effect of K on repairs is minimal (for c equal to 0 or 0.025) or even opposite to Fig 1A-E in S2 (for c=0.05). The effect of K on age at maturity depended on the type of density-dependence and on the type of mortality model (PHM or SSM). The increase in K can slightly accelerate maturity (Fig 1AB in S2) or delay it (Fig 1C-F in S2). This delay is relatively stronger when density-dependence acts on production rate under the highest level of extrinsic mortality (Fig 1CF in S2).

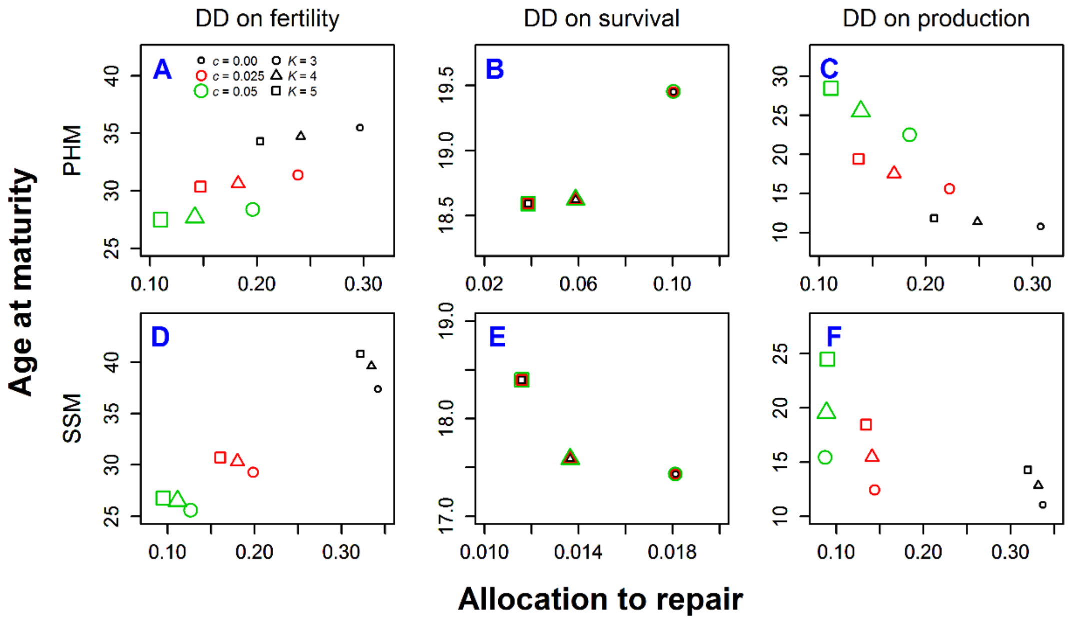

Fig 1. The effect of repair efficiency on evolutionarily stable age at maturity and allocation to repairs. Different types of density-dependence are depicted in columns, different models of the effect of repair on mortality are depicted in rows, different levels of extrinsic mortality are denoted by size and color of the marks, and different repair efficiency K by shapes of marks. All cases of density-dependence are age-independent. The same Gompertz-Makeham parameters are set within the same repair-mortality model: b=0.1 for PHM, and a= –9 for SSM.
