# Supplementary material for: Density-dependence interacts with extrinsic mortality in shaping life histories
Source: PLoS One. 2017 Oct 19;12(10):e0186661. doi: 10.1371/journal.pone.0186661 (PMC5648222; doi:10.1371/journal.pone.0186661)
Supplement: S3 File — (PDF) [file pone.0186661.s003.pdf]

S3. Definition of the shapes of density-dependence used in the paper

In the main text we show how the strength of density-dependence changes with age (Fig 5AEIMQ). The shape of the function describing this relationship is modeled by modifying the Beverton-Holt function (Eq 11) with the power $B(x)$. $B(x)$ is defined as a vector of age-specific weights with values between 0 and 1. This approach is reasonable because it combines the scale and the shape of density-dependence into one inseparable proportional factor Q. If B acted proportionally to Q its effect on Q could not be separated from the effect on the target of density-dependence (e.g. fertility). The general formula for $B(x)$ is :

(1) $$B(x) = h_1 V^{h_2} - V^{h_3} + 1$$

where

(2) $$V(x) = \begin{cases} (x+1)/\omega : h_4 = 0 \\ (\omega - x)/\omega : h_4 = 1 \end{cases}$$

Where ω=120 is the number of age classes in the model, and h_1, h_2, h_3, and h_4 are shape parameters. For the cases represented in Figs 2, 3, and 4 in the manuscript the shape function is 1 for every x (h_1=1, h_2=0, h_3=0, and h_4=1). For case (I) the parameters are set to: h_1=1, h_2=0, h_3=50, and h_4=1; for the case (II) to: h_1=1, h_2=0, h_3=0.5, and h_4=0; and for the case (III) to h_1=2, h_2=0, h_3=2, and h_4=1.
