# Supplementary material for: Density-dependence interacts with extrinsic mortality in shaping life histories
Source: PLoS One. 2017 Oct 19;12(10):e0186661. doi: 10.1371/journal.pone.0186661 (PMC5648222; doi:10.1371/journal.pone.0186661)
Supplement: S4 File — (PDF) [file pone.0186661.s004.pdf]

S4. Algorithm for searching ESS

We solve each case of the model for ESS $\theta^*=\{\tau^*, z^*\}$ by invading consecutive strategies. If the invading strategy wins then it becomes the new resident strategy. In the first round the strategies for such a competition are taken from the set of possible combinations of $\tau^{\mathrm{I}} \in \{ 0, \Delta\tau^{\mathrm{I}}, 2\Delta\tau^{\mathrm{I}},\ldots, 120 - \Delta\tau^{\mathrm{I}}, 120 \}$ and $z^{\mathrm{I}} \in \{ 0, \Delta z^{\mathrm{I}}, 2\Delta z^{\mathrm{I}},\ldots, 1 - \Delta z^{\mathrm{I}}, 1 \}$, where steps in a considered vector are $\Delta\tau^{\mathrm{I}} = 2$ and $\Delta z^{\mathrm{I}} = 0.02$. When all pairs are checked for invasion then we get a first approximation of the ESS strategy $\tau^{*\mathrm{I}}$ and $z^{*\mathrm{I}}$. In the next approximation new vectors of strategies are defined around $\tau^{*\mathrm{I}}$ and $z^{*\mathrm{I}}$, each with a radius that equals 2 times the previous step for a considered vector: $\tau^{\mathrm{II}} \in \{ \tau^{*\mathrm{I}} - 2\Delta\tau^{\mathrm{I}}, \tau^{*\mathrm{I}} - 2\Delta\tau^{\mathrm{I}} + \Delta\tau^{\mathrm{II}},\ldots, \tau^{*\mathrm{I}} + 2\Delta\tau^{\mathrm{I}} - \Delta\tau^{\mathrm{II}}, \tau^{*\mathrm{I}} + 2\Delta\tau^{\mathrm{I}} \}$, where $\Delta\tau^{\mathrm{II}} = 4\Delta\tau^{\mathrm{I}}/10$ is a new step; analogically $z^{\mathrm{II}} \in \{ z^{*\mathrm{I}} - 2\Delta z^{\mathrm{I}}, z^{*\mathrm{I}} - 2\Delta z^{\mathrm{I}} + \Delta z^{\mathrm{II}},\ldots, z^{*\mathrm{I}} + 2\Delta z^{\mathrm{I}} - \Delta z^{\mathrm{II}}, z^{*\mathrm{I}} + 2\Delta z^{\mathrm{I}} \}$, where $\Delta z^{\mathrm{II}} = 4\Delta z^{\mathrm{I}}/10$. Again, all possible pairs are checked for invasion giving the second approximation $\tau^{*\mathrm{II}}$ and $z^{*\mathrm{II}}$. The third, fourth, and fifth approximations are done analogically to the second approximation.
